# Supplementary material for: Feasibility and Safety of Uniportal Thoracoscopic Segmentectomy Using a Unidirectional Dissection Approach without Dissecting a Fissure
Source: Medicina (Kaunas). 2024 Jun 17;60(6):994. doi: 10.3390/medicina60060994 (PMC11205414; doi:10.3390/medicina60060994)
Supplement: Supplementary file 1 [file medicina-60-00994-s001.zip › Supplementary table 1.pdf]

Supplementary Table S1. Comparison of patient characteristics between groups U and C in subset analysis excluding left S1+2, S3, S6 and right S1, S2, S6 segmentectomies.

	Group U (n=17)	Group C(n=49)	p-value
Age, years, median (IQR)	74 (68-83)	71 (66-79)	0.42
Sex			
Female/Male, n (%)	9 (53)/8 (47)	26 (53)/23 (47)	1
Treated lobe			
LUL, n (%)	11 (65)	14 (29)	**0.006**
LLL, n (%)	4 (24)	6 (12)	
RUL, n (%)	1 (6)	10 (20)	
RML, n (%)	0 (0)	0 (0)	
RLL, n (%)	1 (6)	19 (39)	
ASA score, median (IQR)	2 (2-2)	2 (2-2)	0.25
Smoking index, pack x year, median (IQR)	25 (5-50)	15 (0-42.3)	0.20
Preoperative FEV1.0, ml, median (IQR)	1890 (1590-2190)	1860 (1670-2660)	0.45
Preoperative %FEV1.0, %, median (IQR)	82 (78-98)	94 (79-106)	0.32
Disease			
Primary lung cancer, n (%)	12 (70.6)	32 (65.3)	0.65
Pulmonary metastasis, n (%)	3 (17.6)	6 (12.2)	
Other benign, n (%)	2 (11.8)	11 (22.4)	
Kinds of segmentectomy			
Intentional, n (%)	9 (53)	22 (45)	0.55
Unintentional, n (%)	5 (29)	11 (22)	
Others, n (%)	3 (18)	16 (33)	

IQR, interquartile range; LUL, left upper lobe; LLL, left lower lobe; RUL, right upper lobe; RML, right middle lobe; RLL, right lower lobe; ASA, American Society of Anesthesiologists; FEV, forced expiratory volume
