# Supplementary material for: Feasibility and Safety of Uniportal Thoracoscopic Segmentectomy Using a Unidirectional Dissection Approach without Dissecting a Fissure
Source: Medicina (Kaunas). 2024 Jun 17;60(6):994. doi: 10.3390/medicina60060994 (PMC11205414; doi:10.3390/medicina60060994)
Supplement: Supplementary file 1 [file medicina-60-00994-s001.zip › Supplementary table 2.pdf]

Supplementary Table S2. Details of segmentectomies performed in groups U and C in subset analysis excluding left S1+2, S3, S6 and right S1, S2, S6 segmentectomies.

	Group U (n=17)	Group C (n=49)	p-value
Simple/Complex, n (%)	12 (70.6)/5 (29.4)	20 (40.8)/29 (59.2)	0.049
LUL			
S1-3	11	8	
S3-5	0	2	
S4-5	1	4	
LLL			
S8	0	4	
S8-10	0	1	
S9-10	3	0	
S10	0	1	
RUL			
S1+3	0	3	
S3	1	7	
RLL			
S7-8	0	1	
S7-9	0	1	
S7-10	0	7	
S8	0	1	
S8-10	0	2	
S9-10	1	7	
