# Supplementary material for: Feasibility and Safety of Uniportal Thoracoscopic Segmentectomy Using a Unidirectional Dissection Approach without Dissecting a Fissure
Source: Medicina (Kaunas). 2024 Jun 17;60(6):994. doi: 10.3390/medicina60060994 (PMC11205414; doi:10.3390/medicina60060994)
Supplement: Supplementary file 1 [file medicina-60-00994-s001.zip › Supplementary table 3.pdf]

Supplementary Table S3. Comparison of perioperative outcomes between groups U and C in subset analysis excluding left S1+2, S3, S6 and right S1, S2, S6 segmentectomies.

	Group U (n=17)	Group C (n=49)	p-value
Operative time, minutes, median (IQR)	123 (100-130)	151 (120-180)	**0.003**
Blood loss, gram, median (IQR)	0 (0-0)	0 (0-50)	**0.002**
Postoperative drainage time, days, median (IQR)	1 (0-1)	1 (1-1)	0.19
Postoperative drainage time			1
0-1, days (%)	13 (77)	38 (78)	
2-, days (%)	4 (24)	11 (22)	
Postoperative hospitalization time, days, median (IQR)	2.5 (2-4)	3 (2-4)	0.44
Morbidity (Clavien–Dindo classification grade ≥ 3), n (%)	0 (0)	2 (4.1)	1
Readmission within 30 days after discharge, n (%)	0 (0)	0 (0)	—
Conversion to thoracotomy, n (%)	0 (0)	2 (4)	1
30-day mortality, n (%)	0 (0)	1 (2)	1

IQR, interquartile range
